# Dielectric relaxation of high-*k* oxides

**DOI:** 10.1186/1556-276X-8-456

**Published:** 2013-11-01

**Authors:** Chun Zhao, Ce Zhou Zhao, Matthew Werner, Steve Taylor, Paul Chalker

**Affiliations:** 1 Department of Electrical Engineering and Electronics, University of Liverpool, Liverpool L69 3GJ, UK; 2 Department of Electrical and Electronic Engineering, Xi’an Jiaotong-Liverpool University, Suzhou, Jiangsu 215123, China; 3 Department of Engineering, Materials Science and Engineering, University of Liverpool, Liverpool L69 3GH, UK; 4 Nanoco Technologies Ltd, Manchester M13 9NT, UK

**Keywords:** Frequency dispersion, High-*k*, Grain size, Dielectric relaxation

## Abstract

Frequency dispersion of high-*k* dielectrics was observed and classified into two parts: extrinsic cause and intrinsic cause. Frequency dependence of dielectric constant (dielectric relaxation), that is the intrinsic frequency dispersion, could not be characterized before considering the effects of extrinsic frequency dispersion. Several mathematical models were discussed to describe the dielectric relaxation of high-*k* dielectrics. For the physical mechanism, dielectric relaxation was found to be related to the degree of polarization, which depended on the structure of the high-*k* material. It was attributed to the enhancement of the correlations among polar nanodomain. The effect of grain size for the high-*k* materials' structure mainly originated from higher surface stress in smaller grain due to its higher concentration of grain boundary.

## Review

### Background

As the thickness of SiO_2_ gate dielectric films used in complementary metal oxide semiconductor (CMOS) devices is reduced toward 1 nm, the gate leakage current level becomes unacceptable [1-4]. Extensive efforts have been focused on finding alternative gate dielectrics for future technologies to overcome leakage problems [5-7]. Oxide materials with large dielectric constants (so-called high-*k* dielectrics) have attracted much attention due to their potential use as gate dielectrics in metal-oxide-semiconductor field-effect transistor (MOSFETs) [8-12]. Thicker equivalent oxide thickness, to reduce the leakage current of gate oxides, is obtained by introducing the high-*k* dielectric to real application [13-15].

There are a number of high*-k* dielectrics that have been actively pursued to replace SiO_2_. Among them are cerium oxide CeO_2_[16-23], cerium zirconate CeZrO_4_[24], gadolinium oxide Gd_2_O_3_[25-27], erbium oxide Er_2_O_3_[28,29], neodymium oxide Nd_2_O_3_[30,31], aluminum oxide Al_2_O_3_[32,33], lanthanum aluminum oxide LaAlO_3_[34,35], lanthanum oxide La_2_O_3_[36], yttrium oxide Y_2_O_3_[37], tantalum pentoxide Ta_2_O_5_[38], titanium dioxide TiO_2_[39], zirconium dioxide ZrO_2_[40,41], lanthanum-doped zirconium oxide La_*x*_Zr_1*−x*_O_2*−δ*_[42,43], hafnium oxide HfO_2_[44], HfO_2_-based oxides La_2_Hf_2_O_7_[45], Ce_*x*_Hf_*1-x*_O_*2*_[46], hafnium silicate HfSi_*x*_O_*y*_[47], and rare-earth scandates LaScO_3_[48], GdScO_3_[49], DyScO_3_[50], and SmScO_3_[51]. Among them, HfO_2_, HfO_2_-based materials, ZrO_2_, and ZrO_2_-based materials are considered as the most promising candidates combining high dielectric permittivity and thermal stability with low leakage current due to a reasonably high barrier height that limits electron tunneling. CeO_2_ is also proposed to be a possible gate dielectric material, because CeO_2_ has high dielectric constant. CeO_2_ has successfully been added to HfO_2_ in order to stabilize the high-*k* cubic and tetragonal phases. Consequently, La_*x*_Zr_1*−x*_O_2*−δ*_, La_2_Hf_2_O_7_, Ce_*x*_Hf_1*−x*_O_2_, and CeO_2_ have received lots of attention for promising high-*k* gate dielectric materials for potential applications in sub-32-nm node CMOS devices.

Since dielectric relaxation and associated losses impaired MOSFET performance, the larger dielectric relaxation of most high-*k* dielectrics compared with SiO_2_ was a significant issue for their use [52-57]. However, there is insufficient information about dielectric relaxation of high-*k* thin films, which prompts us to investigate the phenomenon and the underlying mechanism. In this paper, the dielectric relaxation of the high-*k* dielectric was reviewed. The extrinsic causes of frequency dispersion during C-V measurement were studied before validating dielectric relaxation. In order to describe dielectric relaxation, many mathematic models were proposed. After mathematical models were finalized for fitting experimental data, physical mechanisms of dielectric relaxation were under investigation. Dielectric relaxation behaviors observed in the high-*k* dielectrics were partly due to the level of stress in the crystalline grains, depending on the grain size, analogous to the behavior of ferroelectric ceramics. As surface stress changes, glasslike transition temperature varied considerably. Dielectric relaxation appears to be a common feature in ferroelectrics associated with non-negligible ionic conductivity.

## Methods

### Sample preparation

HfO_2_, ZrO_2_, and LaAlO_3_ thin films were deposited on n-type Si(100) substrates using liquid injection metal organic chemical vapor deposition (MOCVD) or atomic layer deposition (ALD), carried out on a modified Aixtron AIX 200FE AVD reactor (Herzogenrath, Germany) fitted with the “Trijet”™ liquid injector system. During the MOCVD experiments, oxygen was introduced at the inlet of the reactor. For the ALD experiments, the oxygen was replaced by water vapor, which was controlled by a pneumatic valve. The substrate was rotated throughout all experiments for good uniformity. Auger electron spectroscopy (AES) results suggested they are stoichiometric films. All the high-*k* dielectric layers considered were 16 nm in thickness.

La_*x*_Zr_1*−x*_O_2*−δ*_ thin films were deposited onto n-type Si(100) wafers by the same modified Aixtron AIX 200FE AVD reactor liquid injection ALD at 300°C. Both Zr and La sources were Cp-based precursors ([(MeCp)_2_ZrMe(OMe)] and [(^i^PrCp)_3_La]). The La concentration was varied in different films. Particular attention has been given to the results from films with a La concentration of *x* = 0.09 (55 nm) and *x* = 0.35 (35 nm) but results are also included from films with a concentration of *x* = 0.22 (50 nm) and *x* = 0, i.e., un-doped ZrO_2_ (35 nm). Post deposition annealing was performed at 900°C in a pure N_2_ ambient for 15 min. To form MOS capacitors (Au/La_*x*_Zr_1*−x*_O_2_/IL/n-Si, where IL stands for interfacial layer), metal (Au) gate electrodes with an effective contact area of 4.9 × 10^−4^ cm^2^ were evaporated onto the samples. The backsides of the Si samples were cleaned with a buffered HF solution and subsequently a 200-nm-thick film of Al was deposited by thermal evaporation to form an ohmic back contact.

La_2_Hf_2_O_7_ thin films were deposited on n-type Si(100) substrates by the same liquid injection ALD at 300°C. Both Hf and La sources are Cp-based precursors ([(MeCp)_2_HfMe(OMe)] and [(^i^PrCp)_3_La]). The composition of the La-doped HfO_2_ thin films was estimated to be La_2_Hf_2_O_7_. Selected thin films were subjected to 900°C post-deposition annealing (PDA) in N_2_ for 15 min.

Amorphous Ce_*x*_Hf_1*−x*_O_2_ thin films (*x* = 0.1) were deposited on n-type Si(100) substrates using the same liquid injection ALD. The doping level was varied up to a concentration level of 63%, i.e., *x* = 0.63. The interfacial layer between high*-k* thin film and silicon substrate is approximately 1-nm native SiO_2_. Samples were then annealed at 900°C for 15 min in an N_2_ ambient to crystallize the thin films.

CeO_2_ thin films used the same liquid injection ALD for deposition. The precursor was a 0.05 M solution of [Ce(mmp)4] in toluene and a source of oxygen was deionized water. ALD procedures were run at substrate temperatures of 150, 200, 250, 300, and 350°C, respectively. The evaporator temperature was 100°C and reactor pressure was 1 mbar. CeO_2_ films were grown on n-Si (100) wafers. Argon carrier gas flow was performed with 100 cm^3^ · min^−1^. The flow of [Ce(mmp)_4_]/purge/H_2_O/purge was 2/2/0.5/3.5 s and the number of growth cycles was 300, which is important in order to achieve high reproducibility of film growth and precise control of film thickness by the number of deposition cycles. The thicknesses for the samples are within 56 nm to 98 nm. Post deposition annealing (PDA) was operated on the 250°C as-deposited samples in vacuum at 800°C for 15 min.

### Material characterization

The physical properties of the high-*k* thin films were studied using X-ray diffraction (XRD) and cross-sectional transmission electron microscopy (XTEM). Electrical properties of the films were obtained by capacitance-voltage (C-V) and capacitance-frequency (C-*f*).

XRD were operated using a Rigaku Miniflex diffractometer (Beijing, China) with CuK_α_ radiation (0.154051 nm, 40 kV, 50 mA) spanning a 2*θ* range of 20° to 50° at a scan rate of 0.01°/min.

Atomic force microscopy (AFM) was used to investigate variations in surface morphology of these films, and was carried out using a Digital Instruments Nanoscope III, in contact mode.

AES was used to determine the atomic composition of the thin films, which was carried out using a Varian scanning Auger spectrometer (Palo Alto, CA, USA). The atomic compositions are from the bulk of the thin film, free from surface contamination, and were obtained by combining AES with sequential argon ion bombardment until comparable compositions were obtained for consecutive data points.

XTEM was used to obtain the film thickness and information about the crystal grain size. A JEOL 3010 or a JEOL 2000FX (Akishima-shi, Japan) operated at 300 and 200 keV, respectively, was used.

C-V measurements were implemented using an Agilent E4980A precision LCR meter (Santa Clara, CA, USA). C-V measurements were performed in parallel mode, from strong inversion toward strong accumulation (and vice versa), at frequencies ranging from 20 Hz to 2 MHz. C-*f* measurements were carried out in a strong accumulation region.

## Results and discussion

### Extrinsic frequency dispersion

**Figure 1:**
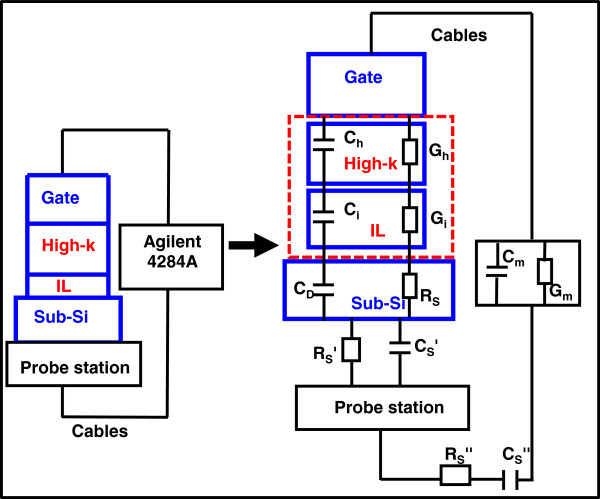
**Causes of frequency dispersion during C-V measurement in the MOS capacitor with high-*k*** **dielectric [**[56]**].**

Frequency dispersion was categorized into two parts: extrinsic causes and intrinsic causes. The extrinsic causes of frequency dispersion during C-V measurement in high-*k* thin film (shown in Figure 1), which were studied before validating the effects of *k*-value dependence, were parasitic effect, lossy interfacial layer, and surface roughness [56]. Two further potential extrinsic causes: polysilicon depletion effect [58-60] and quantum mechanical confinement [61-63], for frequency dispersion were negligible if the thickness of the high-*k* thin film is high enough. Polysilicon depletion effects were not considered due to the implementation of metal gate. Existing causes of extrinsic frequency dispersion during C-V measurement in the high-*k* thin film were the parasitic effect (including back contact imperfection resistance *R*_*S*_^*’*^ and capacitance *C*_*S*_^*”*^, cables resistance *R*_*S*_^*”*^ and capacitance *C*_*S*_^*”*^, substrate series resistance *R*_*S*_*,* and depletion layer capacitance of silicon *C*_*D*_) and the lossy interfacial layer effect (interfacial layer capacitance *C*_*i*_ and conductance *G*_*i*_). Surface roughness effect and polysilicon depletion effect were included, where high-*k* capacitance *C*_*h*_, high-*k* conductance *G*_*h*_, the lossy interfacial layer capacitance *C*_*i*_ and conductance *G*_*i*_ were given. The oxide capacitance *C*_*ox*_ consisted of the high-*k* capacitance *C*_*h*_ and the lossy interfacial layer capacitance *C*_*i*_.

Parasitic effects in MOS devices included parasitic resistances and capacitances such as bulk series resistances, series contact, cables, and many other parasitic effects [64-67]. However, only two of them which had influential importance are listed as follows: (1) the series resistance *R*_*S*_ of the quasi-neutral silicon bulk between the back contact and the depletion layer edge at the silicon surface underneath the gate; and (2) the imperfect contact of the back of the silicon wafer. Dispersion could be avoided by depositing an Al thin film at the back of the silicon substrate. The correction models were able to minimize the dispersion as well. Then, it has been demonstrated that once the parasitic components are taken into account, it was possible to determine the true capacitance values free from errors.

The existence of frequency dispersion in the LaAlO_3_ sample was discussed in the previous work [68], which was mainly due to the effect of the lossy interfacial layer between the high-*k* thin film and silicon substrate on the MOS capacitor. The frequency dispersion effect was significant even with the Al back contact and the bigger substrate area. In this case, *C*_*h*_ (CET = 2.7 nm) was comparable with *C*_*i*_ (approximately 1-nm native SiO_2_) and the frequency dispersion effect was attributed to losses in the interfacial layer capacitance, caused by interfacial dislocation and intrinsic differences in the bonding coordination across the chemically abrupt ZrO_2_/SiO_2_ interface. Relative thicker thickness of the high-*k* thin film than the interfacial layer significantly prevented frequency dispersion. Also, extracted C-V curves were reconstructed by a four-element circuit model for high-*k* stacks, adapted from a dual frequency technique [69], with the capacitance value reconstructed from the loss.

Frequency dispersion from the effect of surface roughness was best demonstrated in an ultra-thin SiO_2_ MOS device [70]. To investigate whether the unwanted frequency dispersion of the high-*k* materials (La_*x*_Zr_1*−x*_O_2*−*δ_) was caused by the surface roughness or not, the surface properties of the La_*x*_Zr_1*−x*_O_2*−δ*_ thin films was studied using AFM [52]. The root mean square (RMS) roughness of the *x* = 0.35 thin film was 0.64 nm after annealing. However, no significant roughness was observed for the *x* = 0.09 thin film (RMS roughness of 0.3 nm). It means that the *x* = 0.35 thin film had more surface roughness than the *x* = 0.09 thin film. The annealed thin film with *x* = 0.09 had large frequency dispersion. However, the annealed thin film with *x* = 0.35 showed small frequency dispersion. By comparing these results from the C-V measurements, it has led to the conclusion that the surface roughness was not responsible for the observed frequency dispersion of the high-*k* dielectric thin films (La_*x*_Zr_1*−x*_O_2*−δ*_).

### Intrinsic frequency dispersion: mathematic models

After careful considerations of the above extrinsic causes for frequency dispersion, high-*k* capacitance *C*_*h*_ was determined. *A* is the area of the MOS capacitance and *t*_*h*_ is the thickness of the high-*k* oxides. Via the equation below, dielectric constant (*k*) was able to be extracted from the high-*k* capacitance.

(1) $$C_{h}=\frac{\mathrm{Ak}\epsilon_{0}}{t_{h}}$$

**Figure 2:**
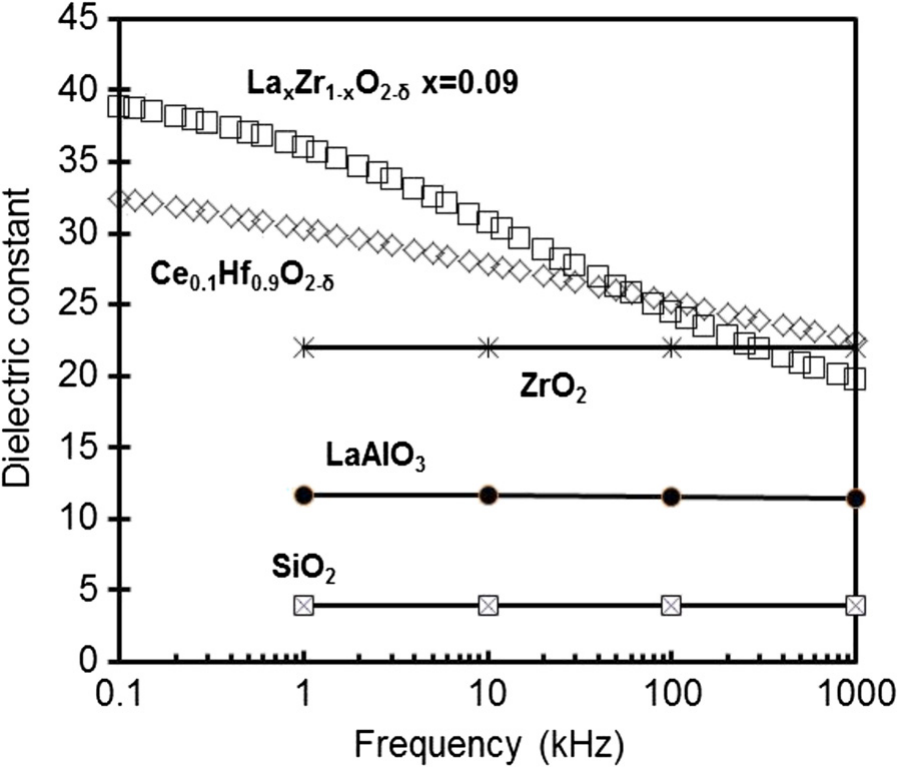
**Frequency dependence of** ***k*** **value extracted from C-*f*** **measurements in the MOS capacitors with high-*k*** **dielectrics [**[52]**,**[55]**,**[56]**].**

Frequency dispersion can now solely be associated with the frequency dependence of the *k*-value. The frequency dependence of the *k* value can be extracted as shown in Figure 2. The figure showed no frequency dependence of the *k* value in LaAlO_3_/SiO_2_, ZrO_2_/SiO_2_ and SiO_2_ stacks [56]. However, the frequency dependence of the *k*-value was observed in La_*x*_Zr_*1–x*_O_2_/SiO_2_ stacks [52]. The zirconium thin film with a lanthanum (La) concentration of *x* = 0.09 showed a sharp decreased *k*-value and suffered from a severe dielectric relaxation. A *k* value of 39 was obtained at 100 Hz, but this value was reduced to a *k* value of 19 at 1 MHz. The 10% Ce-doped hafnium thin film [55] also had a *k* value change from 33 at 100 Hz to 21 at 1 MHz. In order to interpret intrinsic frequency dispersion, many dielectric relaxation models were proposed in terms with frequency dependence of *k* value.

In 1889, the Curie-von Schweidler (CS) law was firstly announced and developed later in 1907 [71,72]. The general type of dielectric relaxation in time domain can be described by the CS law (the *t*^*−n*^ behavior, 0 ≤ *n* ≤ 1).

(2) $$\frac{\mathrm{dP}\left(t\right)}{\mathrm{dt}}\propto t^{-n},$$

where *P(t)* represented the polarization and the exponent *n* indicated the degree of dielectric relaxation. After a Fourier transform, the complex susceptibility CS relation is:

(3) $$\chi_{\mathrm{CS}}=A{\left(\mathrm{i\omega}\right)}^{n-1},$$

where *A* and *n* were the relaxation parameters, *ϵ*_*∞*_ was the high frequency limit of the permittivity, *χ*_*CS*_ *=* [*ϵ*_*CS*_ *×* (*ω*) − *ϵ*_*∞*_]/(*ϵ*_*s*_ − *ϵ*_*∞*_) was the dielectric susceptibility related to the CS law. The value of the exponent (*n*) indicated the degree of dielectric relaxation. The exponent values *n* was a weak dependence of the permittivity on frequency. An *n −* 1 value of zero would indicate that the dielectric permittivity was frequency independent. The majority of the model was based on the presence of compositional or structural inhomogeneities and body effects.

In 1929, Debye described a model for the response of electric dipoles in an alternating electric field [73]. In time domain, the response of the polarization is:

(4) $$\frac{\mathrm{dP}\left(t\right)}{\mathrm{dt}}=-\frac{P\left(t\right)}{\tau }$$

(5) $$P\left(t\right)=P_{0}\;\exp\;\left(-\frac{t}{\tau }\right)$$

Unlike the CS law of power law, Debye law was an equation of exponential. As two main branches in the development of dielectric relaxation modeling, the CS and Debye are the origins along the evolution beyond doubt. The Debye model led to a description for the complex dielectric constant ϵ*. An empirical expression, which originated from the Debye law, was proposed by Kohlrausch, Williams, and Watts, which is a stretched exponential function, to be referred to later as the Kohlrausch-Williams-Watts (KWW) function widely used to describe the relaxation behavior of glass-forming liquids and other complex systems [74-76]. The equivalent of the dielectric response function in time domain is

(6) $$P\left(t\right)=P_{0}\exp\;\left[{\left(-\frac{t}{\tau }\right)}^{\beta_{\mathrm{KWW}}}\right]$$

After a Fourier transform, the Debye equation in the frequency domain and its real and imaginary parts are

(7) $$\epsilon^{*}\left(\omega \right)=\epsilon_{\infty }+\frac{\epsilon_{s}-\epsilon_{\infty }}{1+\left(\mathrm{i\omega \tau}\right)}$$

(8) $$\epsilon^{'}\left(\omega \right)=\epsilon_{\infty }+\frac{\epsilon_{s}-\epsilon_{\infty }}{1+\omega^{2}\tau^{2}}$$

(9) $$\epsilon^{''}\left(\omega \right)\frac{\left(\epsilon_{s}-\epsilon_{\infty }\right)\omega \tau }{1+\omega^{2}\tau^{2}}$$

where *τ* was called the relaxation time which was a function of temperature and it was independent of the time angular frequency *ω* = 2π*f*. *ϵ*_*s*_ was also defined as the zero-frequency limit of the real part, *ϵ’*, of the complex permittivity. *ϵ*_*∞*_ was the dielectric constant at ultra-high frequency. Finally, *ϵ’* was the *k* value.

The Debye theory assumed that the molecules were spherical in shape and dipoles were independent in their response to the alternating field with only one relaxation time. Generally, the Debye theory of dielectric relaxation was utilized for particular types of polar gases and dilute solutions of polar liquids and polar solids. However, the dipoles for a majority of materials were more likely to be interactive and dependent in their response to the alternating field. Therefore, very few materials completely agreed with the Debye equation which had only one relaxation time.

Since the Debye expression cannot properly predict the behavior of some liquids and solids such as chlorinated diphenyl at −25°C and cyclohexanone at −70°C, in 1941, Cole K.S. and Cole R.H. proposed an improved Debye equation, known as the Cole-Cole equation, to interpret data observed on various dielectrics [77]. The Cole-Cole equation can be represented by ϵ*(*ω*):

(10) $$\epsilon^{*}\left(\omega \right)=\epsilon_{\infty }+\frac{\epsilon_{s}-\epsilon_{\infty }}{1+{\left(\mathrm{i\omega \tau}\right)}^{1-\alpha }},$$

where *τ* was the relaxation time and *α* was a constant for a given material, having a value 0 ≤ *α* ≤ 1. *α* = 0 for Debye relaxation. The real and imaginary parts of the Cole-Cole equation are

(11) $$\begin{array}{l} \epsilon^{'}\left(\omega \right)=\epsilon_{\infty }\\ \;+\left(\epsilon_{s}-\epsilon_{\infty }\right)\frac{1+{\left(\omega \tau \right)}^{1-\alpha }\sin\left(\frac{1}{2}\alpha \pi \right)}{1+2{\left(\omega \tau \right)}^{1-\alpha }\sin\left(\frac{1}{2}\alpha \pi \right)+{\left(\omega \tau \right)}^{2\left(1-\alpha \right)}} \end{array}$$

(12) $$\epsilon^{''}\left(\omega \right)=\left(\epsilon_{s}-\epsilon_{\infty }\right)\frac{1+{\left(\omega \tau \right)}^{1-\alpha }\cos\left(\frac{1}{2}\alpha \pi \right)}{1+2{\left(\omega \tau \right)}^{1-\alpha }\sin\left(\frac{1}{2}\alpha \pi \right)+{\left(\omega \tau \right)}^{2\left(1-\alpha \right)}}$$

Ten years later, in 1951, Davidson et al*.* proposed the following expression (Cole-Davidson equation) to interpret data observed on propylene glycol and glycerol [78-81] based on the Debye expression:

(13) $$\epsilon^{*}\left(\omega \right)=\epsilon_{\infty }+\frac{\epsilon_{s}-\epsilon_{\infty }}{{\left(1+\mathrm{i\omega \tau}\right)}^{\beta }},$$

where *τ* was the relaxation time and *β* was a constant for a given material. 0 ≤ *β ≤* 1 which controlled the width of the distribution and *β* = 1 for Debye relaxation. The smaller the value of *β*, the larger the distribution of relaxation times. The real and imaginary parts of the Cole-Davidson equation are given by

(14) $$\epsilon^{'}\left(\omega \right)=\epsilon_{\infty }+\left(\epsilon_{s}-\epsilon_{\infty }\right){\left(\cos\varphi \right)}^{\beta }\cos\beta \varphi$$

(15) $$\epsilon^{''}\left(\omega \right)=\left(\epsilon_{s}-\epsilon_{\infty }\right){\left(\cos\varphi \right)}^{\beta }\sin\beta \varphi$$

(16) $$\varphi =\tan^{-1}\left(\omega \tau \right)$$

Both the Cole-Cole and Cole-Davidson equations were empirical and could be considered to be the consequence of the existence of a distribution of relaxation times rather than that of the single relaxation time (Debye equation). After 15 years, in 1966, S. Havriliak and S. J. Negami reported the Havriliak-Negami (HN) equation which combined the Cole-Cole and Cole-Davidson equations for 21 polymers [82-84]. The HN equation is

(17) $$\epsilon^{*}\left(\omega \right)=\epsilon_{\infty }+\frac{\epsilon_{s}-\epsilon_{\infty }}{{\left[1+{\left(\mathrm{i\omega \tau}\right)}^{1-\alpha }\right]}^{\beta }}$$

**Figure 3:**
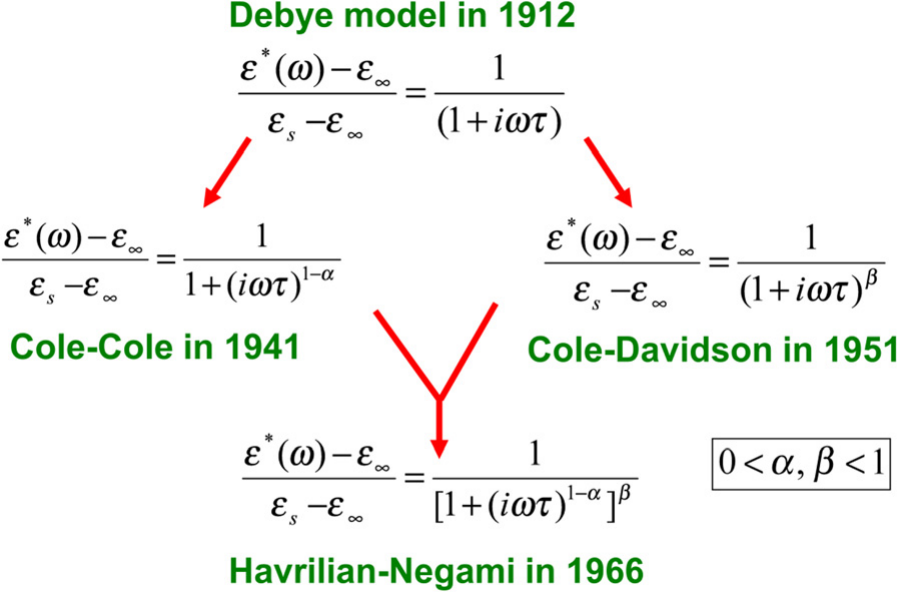
Evolution map for Debye, Cole-Cole, Cole-Davidson, and HN model.

The real and imaginary parts of the HN equation are given by

(18) $$\begin{array}{l} \epsilon^{'}\left(\omega \right)=\epsilon_{\infty }\\ \;+\left(\epsilon_{s}-\epsilon_{\infty }\right)\frac{\cos\left(\beta \Phi \right)}{{\left[1+2{\left(\omega \tau \right)}^{1-\alpha }\sin\left(\frac{\pi \alpha }{2}\right)+{\left(\omega \tau \right)}^{2\left(1-\alpha \right)}\right]}^{\frac{\beta }{2}}} \end{array}$$

(19) $$\epsilon^{''}\left(\omega \right)=\left(\epsilon_{s}-\epsilon_{\infty }\right)\frac{\sin\left(\beta \Phi \right)}{{\left[1+2{\left(\omega \tau \right)}^{1-\alpha }\sin\left(\frac{\pi \alpha }{2}\right)+{\left(\omega \tau \right)}^{2\left(1-\alpha \right)}\right]}^{\frac{\beta }{2}}}$$

(20) $$\Phi =\tan^{-1}\frac{{\left(\omega \tau \right)}^{1-\alpha }\cos\frac{1}{2}\pi \alpha }{1+{\left(\omega \tau \right)}^{1-\alpha }\sin\frac{1}{2}\pi \alpha }$$

where *α* and *β* were the two adjustable fitting parameters. *α* was related to the width of the loss peak and *β* controlled the asymmetry of the loss peak. In this model, parameters *α* and *β* could both vary between 0 and 1. The Debye dielectric relaxation model with a single relaxation time from *α* = 0 and *β* = 1, the Cole-Cole model with symmetric distribution of relaxation times followed for *β* = 1 and 0 ≤ *α* ≤ 1, and the Cole-Davidson model with an asymmetric distribution of relaxation times follows for *α* = 0 and 0 ≤ *β* ≤ 1. The HN equation had two distribution parameters *α* and *β* but Cole-Cole and Cole-Davidson equations had only one. HN model in the frequency domain can accurately describe the dynamic mechanical behavior of polymers, including the height, width, position, and shape of the loss peak. The evolution map for Debye, Cole-Cole, Cole-Davidson, and HN model is shown in Figure 3.

A theoretical description of the slow relaxation in complex condensed systems is still a topic of active research despite the great effort made in recent years. There exist two alternative approaches to the interpretation of dielectric relaxation: the parallel and series models [54]. The parallel model represents the classical relaxation of a large assembly of individual relaxing entities such as dipoles, each of which relaxes with an exponential probability in time but has a different relaxation time. The total relaxation process corresponds to a summation over the available modes, given a frequency domain response function, which can be approximated by the HN relationship.

The alternative approach is the series model, which can be used to describe briefly the origins of the CS law. Consider a system divided into two interacting sub-systems. The first of these responds rapidly to a stimulus generating a change in the interaction which, in turn, causes a much slower response of the second sub-system. The state of the total system then corresponds to the excited first system together with the un-responded second system and can be considered as a transient or meta-stable state, which slowly decays as the second system responds.

In some complex condensed systems, neither the pure parallel nor the pure series approach is accepted and instead interpolates smoothly between these extremes. For the final fitting of the frequency domain response, the frequency dependence of complex permittivity ϵ*(ω) can be combined with the CS law and the modified Debye law (HN law) [52]:

(21) $$\epsilon^{*}\left(\omega \right)=\epsilon_{\infty }+\chi_{\mathrm{CS}}^{*}\left(\omega \right)+\chi_{\mathrm{HN}}^{*}\left(\omega \right)-\frac{i\sigma_{\mathrm{DC}}}{\omega \epsilon_{S}}$$

(22) $$\chi_{\mathrm{CS}}^{*}\left(\omega \right)=A{\left(\mathrm{i\omega}\right)}^{n-1}$$

(23) $$\chi_{\mathrm{HN}}^{*}\left(\omega \right)=\frac{\epsilon_{s}-\epsilon_{\infty }}{{\left[1+{\left(\mathrm{i\omega \tau}\right)}^{1-\alpha }\right]}^{\beta }}$$

where *ϵ*_*∞*_ was the high-frequency limit permittivity, *ϵ*_s_ is the permittivity of free space, *σ*_DC_ is the DC conductivity. The parameters in the equation are in the form of physical meanings (activation energy: *E*_A_):

(24) $$\tau =\tau_{0}\exp\;\left[-\frac{E_{A,\tau }}{k\left(T-T_{\tau }\right)}\right]$$

(25) $$\sigma_{\mathrm{DC}}=\sigma_{0}\exp\;\left[-\frac{E_{A,\sigma }}{k\left(T-T_{\sigma }\right)}\right]$$

(26) $$\alpha =\alpha_{0}\exp\;\left[-\frac{E_{A,\alpha }}{k\left(T-T_{\alpha }\right)}\right]$$

(27) $$\beta =\beta_{0}\exp\;\left[-\frac{E_{A,\beta }}{k\left(T-T_{\beta }\right)}\right]$$

(28) $$n=n_{0}\exp\;\left[-\frac{E_{A,n}}{k\left(T-T_{n}\right)}\right]$$

The HN law was a modified Debye equation via evolution. Thus, the CS and HN laws in the time domain represented the original power-law and exponential dependence, respectively. Most of dielectric relaxation data were able to be modeled by the final fitting law: the combined CS + HN laws.

**Figure 4:**
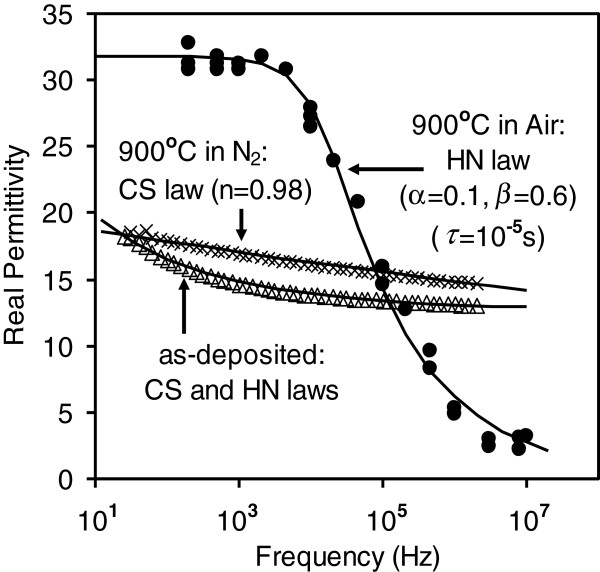
**Dielectric relaxation results of as-deposited and annealed La**_**0.35**_**Zr**_**0.65**_**O**_**2**_**samples [**[54]**].**

Based on the discussion above, the dielectric relaxation results of La_0.35_Zr_0.65_O_2_ for the as-deposited and PDA samples (shown in Figure 4) have been modeled with the CS and/or HN relationships (see solid lines in Figure 4) [54]. The relaxation of the as-deposited film obeyed a combined CS + HN law. After the 900°C PDA, the relaxation behavior of the N_2_-annealed film was dominated by the CS law, whereas the air-annealed film was predominantly modeled by the HN relationship that was accompanied by a sharp drop in the *k* value.

**Figure 5:**
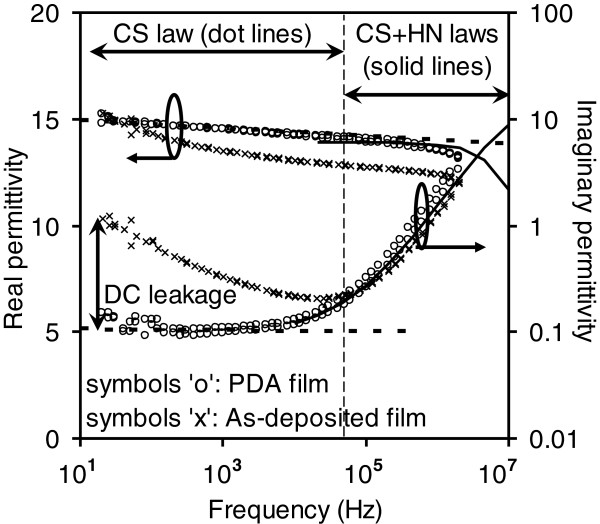
**Dielectric relaxation results in the real and imaginary permittivity of as-deposited and annealed La**_**2**_**Hf**_**2**_**O**_**7**_**samples [**[53]**].**

The frequency-dependent change in the real and imaginary permittivity of La_2_Hf_2_O_7_ dielectric for the as-deposited and PDA samples is shown in Figure 5[53]. Clearly, the PDA process improved the dielectric relaxation and reduced the dielectric loss. The dielectric relaxation of the PDA films was revealed to be dominated mainly by the CS law (*n* = 0.9945, see two dot lines in Figure 5) at *f* < 3 × 10^4^ Hz. However, at *f* > 3 × 10^4^ Hz, the HN law plays an important role (*α* = 0.08, *β* = 0.45, and *τ* = 1 × 10^−8^ s, see two solid lines in Figure 5). The dielectric loss reduces at *f* < 3 × 10^4^ Hz because an increase of the interfacial layer thickness caused the reduction of the DC conductivity.

**Figure 6:**
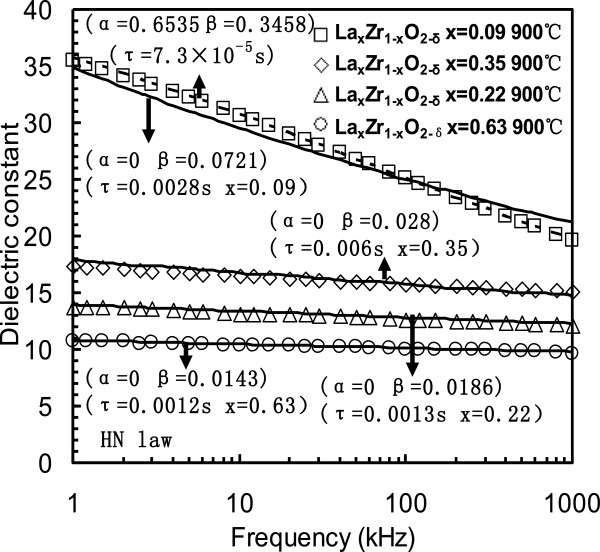
**Dielectric relaxation results of as-deposited La**_***x***_**Zr**_**1** ***−x***_**O**_**2−** ***δ***_**samples [**[56]**].**

Frequency dependence of the *k* value was extracted from C-*f* measurements observed in the La_*x*_Zr_1*−x*_O_2−*δ*_ thin films (shown in Figure 6) [56]. Solid lines are from fitting results from the Cole-Davidson equation, while the dashed line is from the HN equation. The parameters *α*, *β*, and *τ* are from the Cole-Davidson or HN equation. The Cole-Cole and Cole-Davidson equation could fit the dielectric relaxation results of the La_0.91_Zr_0.09_O_2_, La_0.22_Zr_0.78_O_2_, La_0.35_Zr_0.65_O_2_, and La_0.63_Zr_0.37_O_2_ thin films. The La_*x*_Zr_1*−x*_O_2*−δ*_ thin films can be also modeled by the HN equation more accurately than the Cole-Cole and Cole-Davidson equations.

### Intrinsic frequency dispersion: physical mechanisms

**Figure 7:**
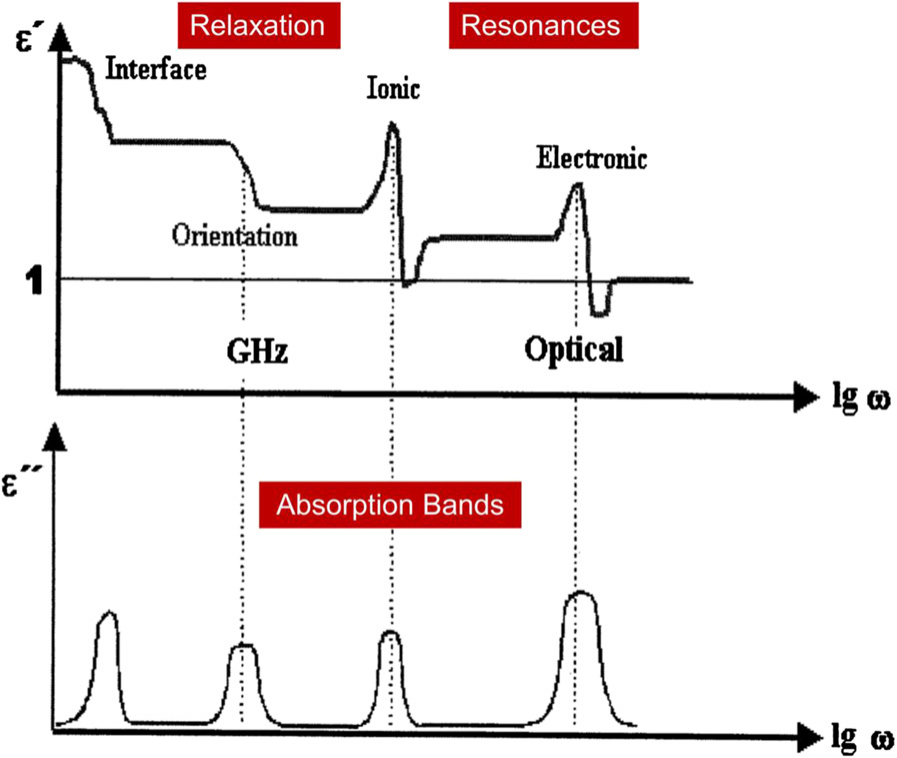
**Physical mechanisms of dielectric relaxation in real and imaginary parts [**[85]**].**

A dielectric material is a non-conducting substance whose bound charges are polarized under the influence of an externally applied electric field. The dielectric behavior must be specified with respect to the time or frequency domain. Different mechanisms show different dynamic behavior in time domain. In consequence, adsorption occurs at different windows in frequency domain. For the physical mechanism of the dielectric relaxation, Figure 7 is to describe the degree of polarization in a given material within frequency domain [85].

The response of the dielectric relaxation in lower frequency range is firstly categorized into the interface polarization. In the region, surfaces, grain boundaries, inter-phase boundaries may be charged, i.e., they contain dipoles which may become oriented to some degree in an external field and thus contribute to the polarization of the material. It is orientation polarization as frequency increasing. Here, the material must have natural dipoles which can rotate freely. As the frequency increases further, dielectric relaxation is termed as ionic and electronic polarization. The mutual displacement of negative and positive sub-lattice in ionic crystals has happened. In this case a solid material must have some ionic character. Then, it is observed that there is displacement of electron shell against positive nucleus. Also, the region is called atomic polarization. In a summary, it is clear that the degree of polarization is related to the structure of the material. In consequence, dielectric behavior in electrostatic and alternating electric fields depends on static and dynamical properties of the structure.

**Figure 8:**
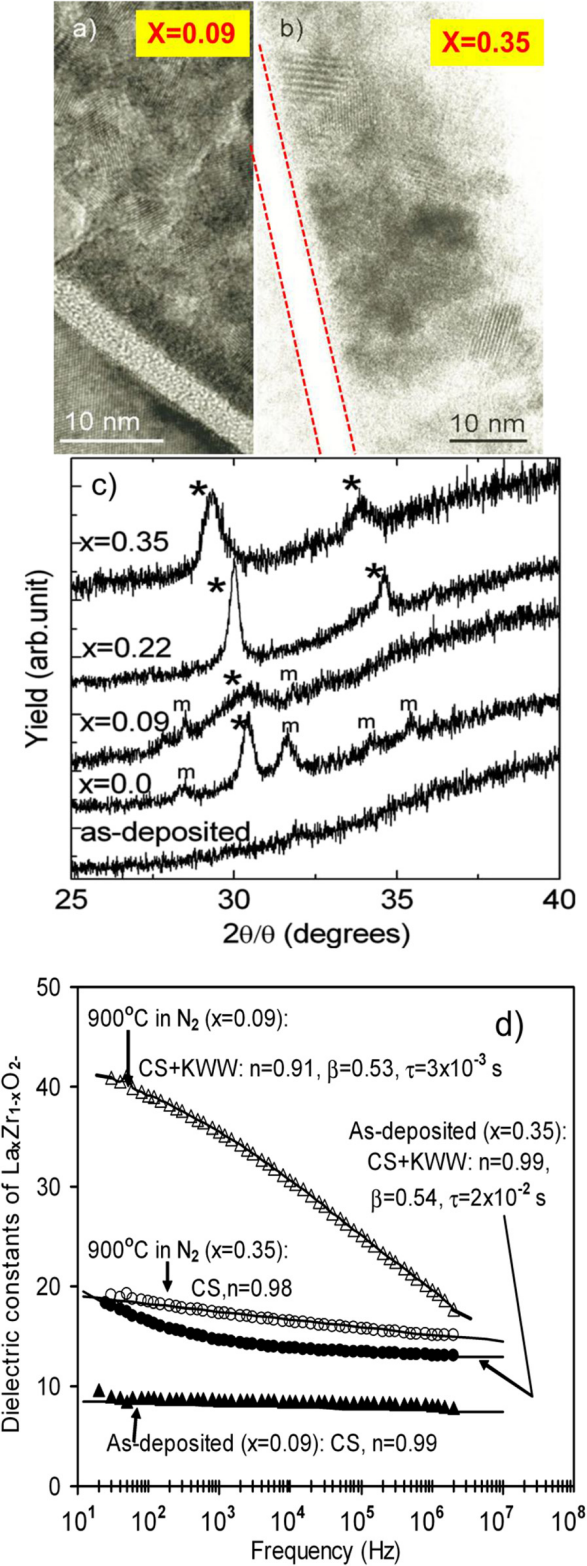
**XTEM (a,b), XRD (c), and k-*f*data (d) of annealed and as-deposited samples. (a)** XTEM of annealed La_0.09_Zr_0.91_O_2_ sample. **(b)** XTEM of annealed La_0.35_Zr_0.65_O_2_ sample. **(c)** XRD of as-deposited La_*x*_Zr_*1−x*_O_2−δ_. **(d)** k-*f* data of as-deposited and annealed La_*x*_Zr_*1−x*_O_2−δ_[52].

XTEM was carried out on both *x* = 0.09 and *x* = 0.35 lanthanum-doped zirconium oxide samples. Images from the annealed samples are shown in Figure 8a,b [52]. These images show that equiaxed nanocrystallites of approximately 4-nm diameter form in the *x* = 0.09 sample, in contrast to a larger crystal of approximately 15-nm diameter for the *x* = 0.35 sample. This trend is also consistent with the average grain size estimated using a Scherrer analysis of the XRD data shown in Figure 8c [52], which gives similar values. In Figure 8d, for the *x* = 0.35 dielectric (open and closed circle symbols), annealing improves the dielectric relaxation and there is less of an effect on the *k* value, i.e., there is a small increase in the *k* value at some frequencies and there is a flatter frequency response compared to the as-deposited sample [52]. The film with a La content of *x* = 0.09 has a significant increase in the *k* value of the dielectric and also has a large dielectric relaxation. For the *x* = 0.09 as-deposited sample, the *k* values are lower and annealing (and hence crystallization into predominantly tetragonal or cubic phase) produces the higher *k* values. It is possible that the dielectric relaxation behavior observed is due to the level of stress in the crystalline grains, depending on the grain size, analogous to the behavior of ferroelectric ceramics.

**Figure 9:**
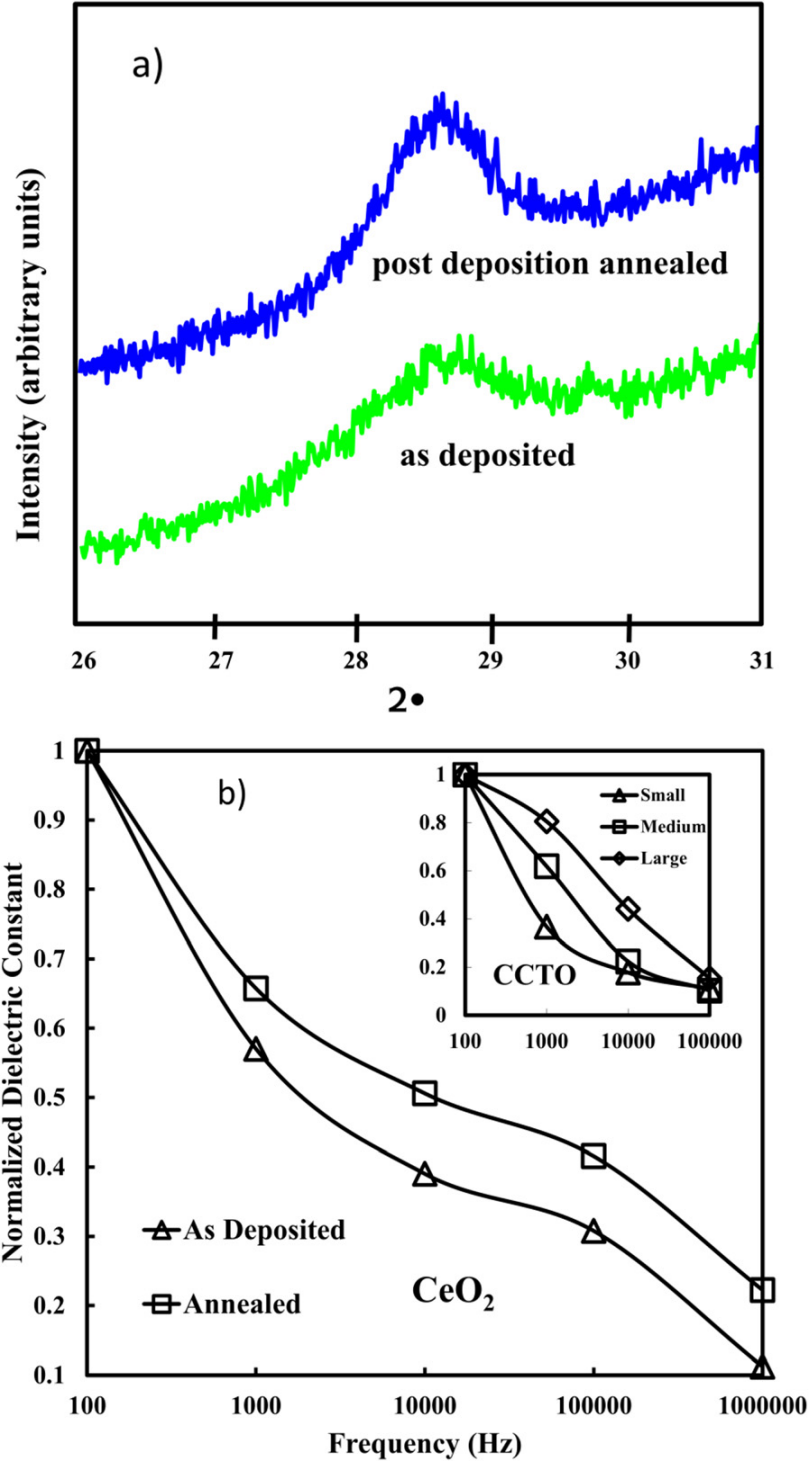
**XRD of (a) and normalized dielectric constants (b) for as-deposited and annealed CeO**_**2**_**samples. (b)** Under different frequencies [57].

An interesting correlation of CeO_2_ as high-*k* thin film between grain size and dielectric relaxation was further discussed afterwards [57]. Figure 9a,b shows XRD diffraction patterns for the as-deposited and annealed samples, respectively. PDA in vacuum at 800°C for 15 min causes an increase in the size of the crystalline grains. The grain size of the annealed sample (9.55 nm) is larger than the original sample (8.83 nm). In order to investigate the frequency dispersion for CeO_2_, normalized dielectric constant in Figure 9b is quantitatively utilized to characterize the dielectric constant variation. It is observed that the dielectric relaxation for the as-deposited sample (triangle symbol) is much serious than the annealed one (square symbol). The smaller the grain size, the more intense is the dielectric relaxation. These findings are in good agreement with the theoretical and experimental studies proposed by Yu et al. [86], which reported the effect of grain size on the ferroelectric relaxor behavior in CaCu_3_TiO_12_ (CCTO) ceramics (shown in inset of Figure 9b). The dielectric relaxation for the small grain size sample is the worst. The effect of grain size mainly originates from higher surface stress in smaller grain due to its higher concentration of grain boundary. Surface stress in grain is high, medium and low for the small, medium, and large grain size CCTO samples. As surface stress increases, the glasslike transition temperature decreases considerably. It is attributed to the enhancement of the correlations among polar nanodomains.

**Figure 10:**
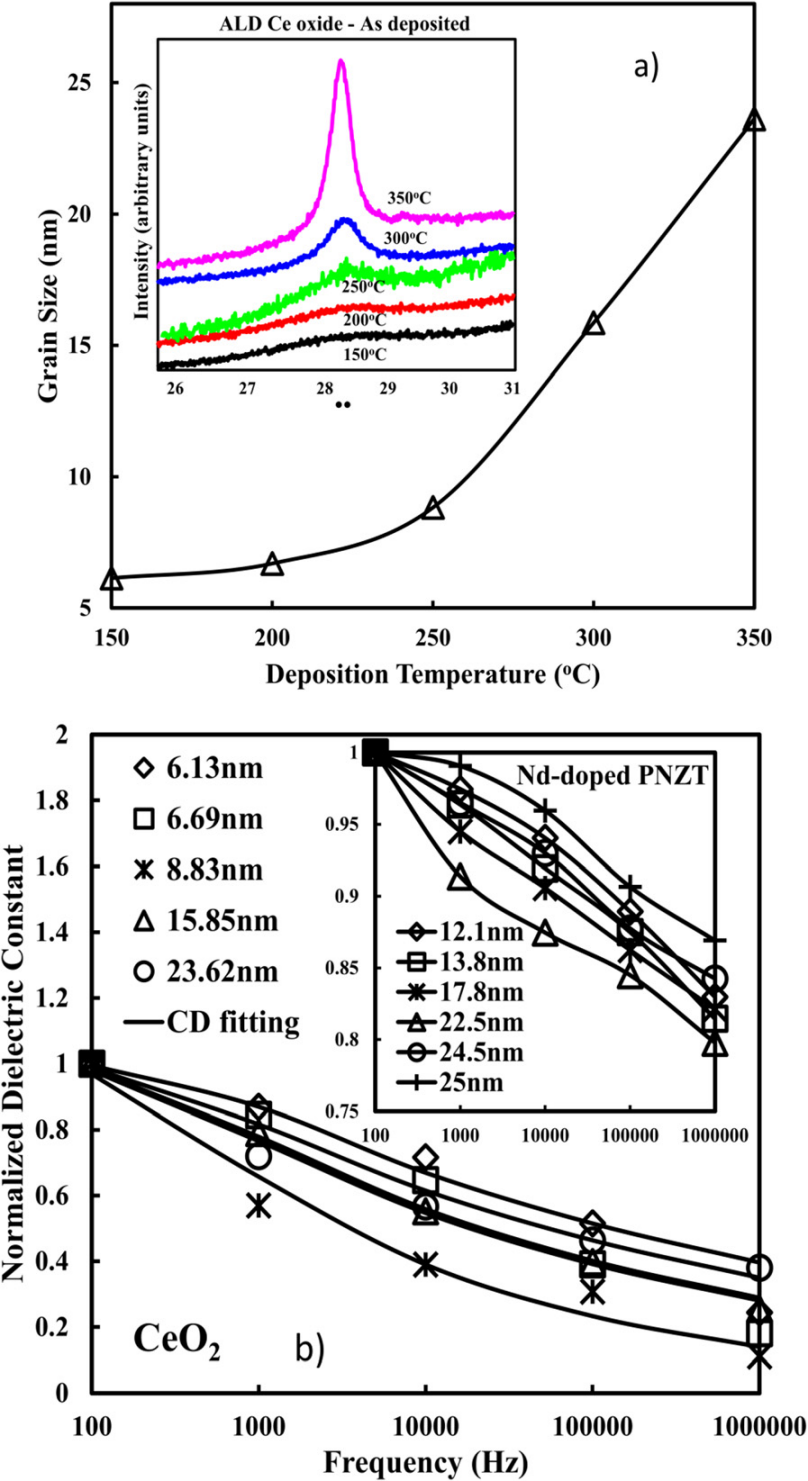
**Grain sizes (a) and normalized dielectric constants (b) for as-deposited CeO**_**2**_**samples. (a)** With various deposition temperatures. **(b)** Under different frequencies [57].

XRD diffraction patterns for the as-deposited CeO_2_ thin films at 150, 200, 250, 300, and 350°C, respectively, are shown in the inset of Figure 10a [57]. The grain size value is obtained in Figure 10a using the Scherrer formula based on the XRD data. There is a clear trend that the grain size increases with increasing deposition temperatures. In Figure 10b, large dielectric relaxation is observed for the sample of 6.13 nm (diamond symbol) [57]. When the deposition temperature increases, the dielectric relaxation is even worse for the sample of 6.69 nm (square symbol). In addition, the most severe dielectric relaxation is measured for the sample of 8.83 nm (star symbol). The sample of 15.85 nm (triangle symbol) has significant improvement on the dielectric relaxation and the sample of 23.62 nm (round symbol) shows more stable frequency response. Similarly, the effect of grain size on the dielectric relaxation is found on the Nd-doped Pb_1−3*x/*2_Nd_*x*_(Zr_0.65_Ti_0.35_)O_3_ composition (PNZT) [87], where *x* = 0.00, 0.01, 0.03, 0.05, 0.07, and 0.09, respectively. It is observed in the inset of Figure 10b that the deteriorative degree of dielectric relaxation increases from 12.1 nm, reaches the peak at 22.5 nm, and then declines. One possible reason for the observation above could be due to the broadened dielectric peak and the transition temperature shift. The transition temperature of PNZT samples is found to shift forward to lower temperature with the grain size from 12.1 to 22.5 nm, while the transition temperature remains at the same position with further increasing grain size. Such strong frequency dispersion in the dielectric constant appears to be a common feature in ferroelectrics associated with non-negligible ionic conductivity.

## Conclusions

In C-V measurements, frequency dispersion in high-*k* dielectrics is very common to be observed. Dielectric relaxation, that is the intrinsic frequency dispersion, could not be assessed before suppressing the effects of extrinsic frequency dispersion. The dielectric relaxation models in the time domain (such as the Debye law and the CS law) and in the frequency domain after the Fourier transform (such as the Cole-Cole equation, the Cole-Davidson equation, the HN equation) were comprehensively considered. The relationship between the grain size and dielectric relaxation is observed in lanthanum-doped zirconium oxide samples. The mechanisms of grain size effects for CeO_2_ are discussed accordingly. A similar relationship between the grain size and dielectric relaxation is also found in CCTO and Nd-doped PNZT samples. The mechanism is attributed to the alignment enhancement of the polar nanodomains.

## Competing interests

The authors declare that they have no competing interests.

## Authors' contributions

CZ reviewed the data and drafted the manuscript. CZZ lead the experiments and supervised the project. MW prepared the samples and performed the characterization. ST and PC participated in the discussions. All authors read and approved the final manuscript.

## Authors’ information

CZ is a PhD student in the University of Liverpool. CZZ is a professor in Xi'an Jiaotong-Liverpool University. MW is a scientist in Nanoco Technologies Ltd. ST and PC are professors in the University of Liverpool.
